# 787 Two Year Follow Up of Microblading in Patients with a Facial Burn Injury

**DOI:** 10.1093/jbcr/irac012.338

**Published:** 2022-03-23

**Authors:** Melissa Brown, Lori Turgeon, Branko Bojovic

**Affiliations:** Shriners Hospitals for Children - Boston, Boston, Massachusetts; Shriners Hospitals for Children - Boston, Boston, Massachusetts; Massachusetts General Hospital/Shriners Hospitals for Children-Boston, Boston, Massachusetts

## Abstract

**Introduction:**

Recovery from a facial burn injury can be challenging. Excessive reconstructive interventions and the emotional devastation can be taxing. Microblading, semi-permanent eyebrow tattooing, is a potential non-invasive alternative treatment. Little research exists on microblading’s longevity and durability over burn scar or its impact on self-esteem. A single case study showed positive impact on patient perceived body image and maintenance of shape and color. Two years post microblading new data supports its efficacy as a potential treatment for patients with a facial burn.

**Methods:**

Single case study two years post microblading of a 22-year-old female who sustained a 30% total body surface area burn injury to bilateral upper extremities, hands and face at age one. Based on referral from her plastic surgeon, she underwent first microblading treatment March 2019 with a standard touch up session May 2019. Patient has not undergone any additional sessions since. Photos obtained two years post treatment were compared to before/after photos immediately following microblading to assess longevity and durability on scar tissue. Self-reported patient outcomes obtained two years after microblading assessed impact on self-esteem and body image.

**Results:**

Photos were taken pre/post microblading, at nine months, and 2.5 years post. Comparison of photos over time showed some expected fading but maintenance of overall shape. Patient self-reports using an eyebrow pencil to darken when she desires brows that are more vibrant and trims hair as needed to maintain shape. Patient reports positive impact to her self-esteem post-microblading. “I most definitely did notice some changes in my self-esteem.” She notes feeling happier and more confident, especially in social situations with peers. Comparison of responses immediately post-microblading with 2.5 years later show focus on improved self-esteem versus satisfaction with appearance of the eyebrow.

**Conclusions:**

Comparison of follow up photos obtained 2.5 years after initial microblading shows maintenance of eyebrow shape with some fading of color. Further research is needed to determine if color fading is as expected or more pronounced in patients with scarred skin. Also important is the impact of characteristic dryness of scars on fading rate. To maintain adequate color, additional treatments would be recommended. Better understanding of factors affecting fading would help determine frequency and timing of maintenance sessions. Patient report indicates increasingly improved self-esteem over time as evidenced by focus on confidence versus appearance. Despite some fading, data supports microblading as an effective non-surgical treatment in establishing eyebrow appearance and function over burn scar.

